# Subclinical Inflammation in Ischemic Heart Disease and Its Role in the Transition to Heart Failure

**DOI:** 10.3390/life16050789

**Published:** 2026-05-08

**Authors:** Costin Petru Groza, Ovidiu Oita, Radu Sebastian Gavril, Oana Irina Gavril, Tatiana Dramba, Ionica Grigore, Cristina Strobescu-Ciobanu, Roxana Nemtanu, Irina Mihaela Esanu

**Affiliations:** 1 Grigore T. Popa University of Medicine and Pharmacy Iasi, 700115 Iași, Romania; costin-petru.groza@d.umfiasi.ro (C.P.G.); sebastian.gavril@umfiasi.ro (R.S.G.); oana-irina.gavril@umfiasi.ro (O.I.G.); tatiana.dramba@email.umfiasi.ro (T.D.); strobescu@yahoo.com (C.S.-C.); roxana.maxim@umfiasi.ro (R.N.); irina.esanu@umfiasi.ro (I.M.E.); 2 “Dunarea de Jos” University of Medicine and Pharmacy, 800008 Galati, Romania; ionica.grigore@ugal.ro

**Keywords:** ischemic cardiomyopathy, subclinical inflammation, heart failure progression, myocardial remodeling, biomarkers, Galectin-3, CA125, high-sensitivity C-reactive protein, precision medicine

## Abstract

Ischemic heart disease (IHD) remains the leading cause of chronic heart failure (HF) worldwide, yet the biological processes underlying this transition are not fully elucidated. Growing evidence indicates that chronic, low-grade inflammation acts as a pivotal link between ischemic injury and progressive myocardial dysfunction. Our review is the most up-to-date and structured synthesis on the pathophysiological pathways, biomarkers, and therapeutic implications of subclinical inflammation in patients with IHD at risk of developing HF. Following acute or repetitive ischemic episodes, persistent immune activation—mediated through interleukin-6 (IL-6), interleukin-1β (IL-1β), and tumor necrosis factor-α (TNF-α)—promotes endothelial dysfunction, microvascular instability, and extracellular matrix remodeling. These mechanisms culminate in ventricular stiffness, diastolic impairment, and adverse structural remodeling, even when left ventricular ejection fraction is preserved. Biomarkers such as Galectin-3, cancer antigen 125 (CA125), and high-sensitivity C-reactive protein (hsCRP) provide valuable insight into the interplay between fibrosis, congestion, and systemic inflammatory load, supporting early detection of subclinical myocardial injury. Advanced imaging modalities, including strain echocardiography and cardiac magnetic resonance imaging (MRI) mapping, enhance the phenotypic characterization of inflammatory cardiomyopathy. Understanding and targeting these inflammatory pathways may open new avenues for precision-based prevention and treatment, ultimately improving outcomes across the IHD–HF continuum.

## 1. Introduction

Ischemic heart disease (IHD) remains the most prevalent cardiovascular condition worldwide and continues to represent a leading cause of heart failure (HF). Despite substantial advances in reperfusion strategies, pharmacological therapies, and preventive cardiology, a considerable proportion of patients experiencing myocardial ischemia ultimately progress to chronic heart failure. The transition from ischemic myocardial injury to ventricular dysfunction is a complex and multifactorial process involving cardiomyocyte death, neurohormonal activation, microvascular dysfunction, and remodeling of the extracellular matrix (ECM) [1,2].

In recent years, increasing attention has been directed toward the role of chronic low-grade inflammation in this pathological progression. Although the inflammatory response triggered by acute myocardial injury initially contributes to tissue repair and scar formation, persistent activation of inflammatory pathways may become maladaptive. Sustained inflammatory signaling promotes adverse cardiac remodeling, stimulates myocardial fibrosis, and contributes to the progressive deterioration of cardiac function [1,3].

Several inflammatory mediators have been implicated in this process. Among these, interleukin-1β (IL-1β), interleukin-6 (IL-6), and tumor necrosis factor-α (TNF-α) represent key cytokines involved in the transition from ischemic injury to structural and functional myocardial remodeling. These mediators exert multiple detrimental effects, including endothelial dysfunction, increased oxidative stress, reduced nitric oxide bioavailability, and accelerated extracellular matrix turnover [3,4]. Importantly, inflammatory activation may occur even in patients with preserved left ventricular ejection fraction (LVEF), suggesting that subclinical inflammation can precede the onset of overt systolic dysfunction [5].

Simultaneously, advances in cardiovascular imaging and biomarker research have substantially improved the detection of early myocardial injury and subtle inflammatory activation. Circulating biomarkers such as galectin-3, high-sensitivity C-reactive protein (hsCRP), and cancer antigen 125 (CA125) have emerged as valuable indicators of myocardial fibrosis, systemic inflammation, and hemodynamic congestion [2,4]. In parallel, modern imaging modalities—including speckle-tracking strain echocardiography and cardiac magnetic resonance (CMR) with parametric mapping—provide detailed insights into myocardial structure, function, and tissue composition, enabling earlier identification of subclinical myocardial alterations [3].

Collectively, these findings suggest that subclinical inflammation represents a critical pathophysiological link between ischemic heart disease and the subsequent development of heart failure. A better understanding of the molecular and cellular mechanisms underlying this relationship may improve early diagnostic strategies and support the development of targeted therapeutic approaches aimed at preventing the progression from ischemic myocardial injury to chronic heart failure [1,3,6].

Therefore, the aim of this review is to summarize current evidence regarding the role of subclinical inflammation in ischemic heart disease and its contribution to the transition toward heart failure. Particular attention is given to inflammatory cytokines, extracellular matrix remodeling, emerging biomarkers, and advanced imaging techniques that may facilitate early detection of inflammatory myocardial injury and adverse cardiac remodeling.

## 2. Materials and Methods

A comprehensive literature search was conducted using the PubMed, Scopus, and Web of Science databases from their inception until 12 December 2025. Articles published between 2000 and 2025 were considered, with particular emphasis placed on studies published during the last 10–15 years, in order to ensure the inclusion of the most recent developments in cardiovascular inflammation and heart failure research.

The search strategy included a combination of relevant keywords and Medical Subject Headings (MeSH) terms. The following search terms were used in different combinations: ischemic heart disease, heart failure, subclinical inflammation, chronic inflammation, interleukin-6, galectin-3, CA125, high-sensitivity C-reactive protein, cardiac remodeling, cardiac magnetic resonance, and strain echocardiography. Boolean operators (AND, OR) were applied to optimize the search strategy and increase the sensitivity of article retrieval.

Studies were considered eligible if they fulfilled the following inclusion criteria: (1) Investigated the association between ischemic heart disease and the development or progression of heart failure. (2) Evaluated inflammatory pathways involved in myocardial injury, fibrosis, or ventricular remodeling. (3) Reported data on inflammatory biomarkers relevant to cardiovascular disease, including cytokines and fibrosis-related markers. (4) Discussed the use of advanced cardiac imaging techniques for detecting myocardial inflammation, fibrosis, or structural remodeling. (5) Were published as original research articles, randomized clinical trials, observational studies, or systematic reviews. (6) Were written in English.

Studies were excluded if they lacked methodological rigor, did not address inflammatory mechanisms in cardiovascular disease, or were limited to case reports or conference abstracts. All identified records were initially screened based on titles and abstracts to determine their relevance to the research topic. Full-text articles were subsequently evaluated when the abstract indicated potential eligibility. Particular emphasis was placed on large clinical trials, prospective cohort studies, translational investigations, and mechanistic studies exploring inflammatory pathways in ischemic heart disease and heart failure.

Following the literature selection process, a total of 68 unique articles were included in this review. These comprised 24 review articles, 37 clinical and observational studies, 5 experimental or translational investigations, and 2 consensus statements relevant to inflammation, biomarkers, and imaging in ischemic heart disease and heart failure.

After the selection process, the studies were categorized according to the main thematic areas addressed in this narrative review: (1) Inflammatory mechanisms linking ischemic heart disease and heart failure. (2) Key inflammatory biomarkers involved in myocardial remodeling and disease progression. (3) Role of advanced cardiac imaging in detecting inflammatory myocardial injury.

This methodological approach allowed for a structured synthesis of current evidence regarding the role of subclinical inflammation as a pathophysiological bridge between ischemic heart disease and heart failure, highlighting both mechanistic insights and emerging diagnostic and therapeutic perspectives.

The authors used generative artificial intelligence tools to assist in graphical illustration design, and the synthesis and interpretation of information derived from previously published studies. All outputs generated with AI support were carefully reviewed, verified, and approved by the authors, who take full responsibility for the final content of the manuscript.

## 3. Results

### 3.1. Pathophysiological Mechanisms Linking Ischemia and Inflammation

#### 3.1.1. The Inflammatory Response Following Myocardial Ischemia

Myocardial ischemia initiates a complex cascade of cellular and molecular events aimed at removing injured tissue and promoting myocardial repair. Immediately following ischemic injury, the innate immune system is activated, triggering an inflammatory response that plays a central role in regulating cardiac healing and remodeling processes [1,7,8,9].

During the early phase of myocardial injury, damaged cardiomyocytes release intracellular molecules known as damage-associated molecular patterns (DAMPs). These signals activate pattern-recognition receptors expressed by endothelial cells and resident immune cells, thereby initiating the recruitment of circulating inflammatory cells to the injured myocardium [10]. Among these, neutrophils are the first to accumulate at the site of injury, where they contribute to the clearance of necrotic tissue through the release of proteolytic enzymes, reactive oxygen species, and pro-inflammatory mediators that facilitate degradation of damaged extracellular matrix components [11].

As the inflammatory response progresses, circulating monocytes infiltrate the myocardium and differentiate into macrophages, which orchestrate both the inflammatory and reparative phases of tissue healing. These macrophages secrete a wide range of cytokines and chemokines, including interleukin-1β (IL-1β), interleukin-6 (IL-6), and tumor necrosis factor-α (TNF-α). Through these mediators, macrophages amplify the inflammatory response and promote further leukocyte recruitment to the injured myocardium [12,13,14]. Although this response is essential for effective clearance of damaged tissue and initiation of repair mechanisms, excessive or prolonged inflammatory activation may contribute to persistent myocardial injury and adverse ventricular remodeling [15].

Ischemic injury also affects the vascular endothelium, leading to increased expression of adhesion molecules such as intercellular adhesion molecule-1 (ICAM-1) and vascular cell adhesion molecule-1 (VCAM-1). These molecules facilitate leukocyte adhesion and transmigration into myocardial tissue, thereby further amplifying the local inflammatory response [16,17]. In this pro-inflammatory environment, activated cardiac fibroblasts begin to synthesize extracellular matrix proteins, promoting fibrotic remodeling and contributing to increased myocardial stiffness.

In many patients with ischemic heart disease, particularly those with stable coronary artery disease or coronary microvascular dysfunction, ischemia may occur recurrently over time. These repeated ischemic episodes can sustain a state of chronic low-grade inflammation within the myocardium. Unlike the intense inflammatory response observed following acute myocardial infarction, this persistent inflammatory activity is more subtle but continuous and may progressively impair endothelial function and coronary microcirculation [2,3,18]. Over time, chronic inflammation contributes to progressive myocardial fibrosis, deterioration of ventricular compliance, and ultimately the development of heart failure [19,20].

Taken together, these mechanisms highlight the pivotal role of inflammation as a biological link between ischemic myocardial injury and the progressive structural and functional changes that characterize heart failure. Increasing evidence supports the concept that persistent inflammatory activation is not merely a consequence of ischemic damage but an active driver of long-term cardiac dysfunction [13,21,22].

#### 3.1.2. Cytokine-Mediated Signaling Pathways

Inflammatory cytokines play a central role in mediating the biological response to myocardial injury and represent key regulators of the transition from ischemic damage to structural and functional cardiac remodeling. Among the numerous inflammatory mediators implicated in cardiovascular disease, interleukin-1β (IL-1β), interleukin-6 (IL-6), and tumor necrosis factor-α (TNF-α) are among the most extensively studied due to their critical involvement in immune activation and myocardial remodeling processes [7,23].

Interleukin-1β is considered a primary initiator of the inflammatory cascade following myocardial ischemia. This cytokine is released predominantly by activated macrophages and other immune cells in response to tissue injury and contributes to the recruitment and activation of leukocytes within the damaged myocardium. Through amplification of downstream cytokine signaling pathways, IL-1β sustains and propagates the inflammatory response beyond the initial phase of injury [24]. Both experimental and clinical studies have demonstrated that persistent IL-1β signaling is associated with myocardial fibrosis, disruption of calcium homeostasis, and impaired contractile function, thereby facilitating the development of ventricular dysfunction [25].

Interleukin-6 represents another key mediator in the inflammatory response associated with ischemic heart disease. As a pleiotropic cytokine, IL-6 is involved in immune regulation, activation of the acute-phase response, and modulation of metabolic pathways. Elevated circulating levels of IL-6 have been consistently associated with increased cardiovascular risk, including a higher incidence of heart failure and adverse cardiac events [26,27]. In addition to its systemic effects, IL-6 can directly influence myocardial remodeling by promoting fibroblast activation and enhancing extracellular matrix deposition, processes that contribute to increased myocardial stiffness and progressive ventricular dysfunction [28].

Tumor necrosis factor-α also plays a pivotal role in the pathophysiology of ischemic myocardial injury. Initially identified as a major mediator of systemic inflammation, TNF-α exerts multiple deleterious effects on cardiac tissue. Experimental studies have shown that TNF-α can induce negative inotropic effects in cardiomyocytes, impair mitochondrial function, and increase oxidative stress within the myocardium [29]. Furthermore, chronic activation of TNF-α signaling pathways promotes cardiomyocyte apoptosis and contributes to adverse ventricular remodeling, thereby accelerating the progression from ischemic injury to overt heart failure [19].

Collectively, these cytokine-mediated mechanisms illustrate how persistent inflammatory signaling influences both cellular and structural components of myocardial remodeling. A deeper understanding of the complex interactions among these inflammatory mediators has become a major focus of contemporary cardiovascular research, particularly in the context of developing targeted therapeutic strategies aimed at modulating inflammation in ischemic heart disease [7,30].

#### 3.1.3. Endothelial Dysfunction and Microvascular Impairment

Endothelial dysfunction represents a key pathophysiological link between chronic inflammation and myocardial injury in patients with ischemic heart disease. The vascular endothelium plays a central role in maintaining cardiovascular homeostasis by regulating vascular tone, controlling leukocyte adhesion, and modulating inflammatory responses. However, persistent inflammatory activation disrupts these protective mechanisms and leads to significant alterations in endothelial function [31,32].

Pro-inflammatory cytokines such as interleukin-1β, interleukin-6, and tumor necrosis factor-α interfere with the synthesis and bioavailability of nitric oxide (NO), a critical mediator of vascular relaxation and endothelial integrity. Reduced nitric oxide availability, together with increased oxidative stress, results in impaired vasodilation and promotes a pro-inflammatory and pro-thrombotic endothelial phenotype [33]. In addition, inflammatory mediators increase endothelial permeability and facilitate the adhesion and transmigration of circulating leukocytes into the vascular wall, thereby further amplifying local inflammation [34].

These alterations have a profound impact on coronary microvascular function. Coronary microcirculation is essential for maintaining adequate myocardial perfusion, particularly under conditions of increased metabolic demand. When endothelial function is compromised, the ability of the microvasculature to appropriately regulate blood flow is impaired, leading to reduced myocardial perfusion and increased susceptibility to ischemia, even in the absence of significant epicardial coronary artery disease [35].

Chronic inflammation within the microvasculature may also induce structural alterations at the level of the myocardial capillary network. These changes include endothelial swelling, inflammatory cell infiltration, and progressive loss of capillary density, a process known as capillary rarefaction [36]. Collectively, these alterations impair oxygen delivery to myocardial tissue and may result in myocardial edema and persistent tissue hypoxia.

These mechanisms appear to be particularly relevant in patients with heart failure and preserved ejection fraction (HFpEF). In this population, microvascular dysfunction and endothelial inflammation are thought to precede overt systolic impairment and contribute to increased myocardial stiffness and impaired diastolic relaxation [37,38]. Consequently, the interplay between systemic inflammation and coronary microvascular dysfunction has emerged as a key determinant in the progression from ischemic heart disease to heart failure, particularly in patients with relatively preserved systolic function.

**Figure 1:**
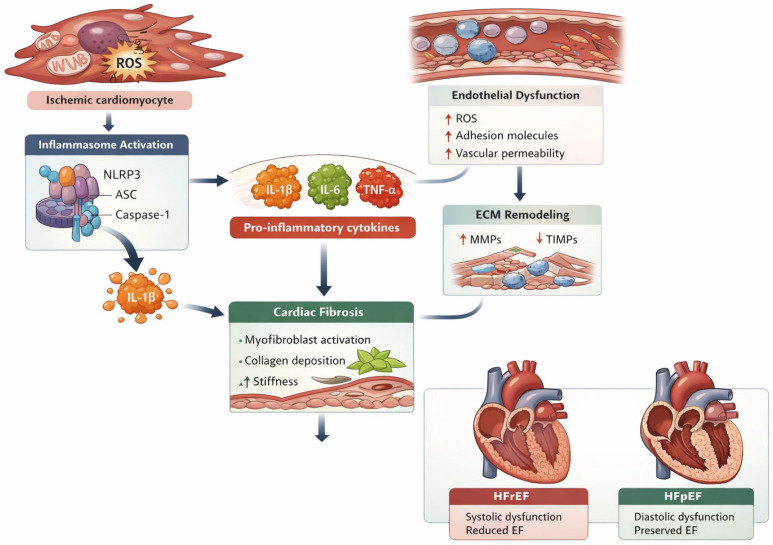
Inflammation-driven transition from ischemic heart disease to heart failure.

The key mechanisms linking inflammation to endothelial dysfunction and microvascular impairment are summarized in Figure 1.

### 3.2. Extracellular Matrix Remodeling and Myocardial Fibrosis

The extracellular matrix (ECM) plays a fundamental role in maintaining myocardial structural integrity and providing the mechanical scaffold required for cardiomyocyte function. Following ischemic injury, inflammatory signaling triggers activation of cardiac fibroblasts and promotes extracellular matrix remodeling through the tightly regulated synthesis and degradation of structural proteins. Matrix metalloproteinases (MMPs) are central regulators of this process, mediating the degradation of ECM components and contributing to myocardial remodeling in both acute and chronic cardiac injury [39,40]. In the early phases following myocardial infarction, inflammatory activation stimulates fibroblast proliferation and accelerates ECM turnover, facilitating scar formation and structural stabilization of the injured myocardium [41].

Although a controlled fibrotic response is essential for preserving myocardial integrity after infarction, excessive or sustained fibroblast activation leads to pathological remodeling characterized by increased collagen deposition and myocardial stiffening. Myofibroblast differentiation represents a key mechanism driving this process and contributes to adverse ventricular remodeling and progressive cardiac dysfunction [42]. Diffuse interstitial fibrosis and alterations in extracellular matrix composition are increasingly recognized as central determinants of impaired myocardial relaxation and the development of heart failure with preserved ejection fraction (HFpEF) [43].

Inflammatory signaling pathways exert a major influence on ECM turnover by modulating fibroblast activity and disrupting the balance between MMPs and their regulatory mechanisms. Persistent inflammatory activation promotes maladaptive remodeling characterized by progressive ventricular dilation, myocardial fibrosis, and deterioration of mechanical function [40]. These structural alterations reduce myocardial compliance and contribute to the development of ventricular dysfunction, as well as to the formation of arrhythmogenic substrates in advanced stages of heart disease.

Recent advances in circulating biomarker assessment have improved the detection and monitoring of myocardial fibrosis and remodeling. Biomarkers such as soluble ST2 and galectin-3 reflect myocardial stress, fibrosis, and inflammatory activation and have demonstrated significant prognostic value in patients with heart failure [44,45,46]. In addition, markers associated with congestion and systemic inflammatory activation, such as CA125, have been shown to correlate with disease severity and long-term clinical outcomes in heart failure populations [47,48].

Advanced imaging techniques further complement biomarker assessment in the identification of early myocardial remodeling. In particular, speckle-tracking echocardiography and the evaluation of global longitudinal strain (GLS) enable the detection of subclinical myocardial dysfunction before significant reductions in left ventricular ejection fraction occur [49]. The integration of circulating biomarkers with advanced imaging modalities therefore represents a promising strategy for the early identification of patients at risk of maladaptive remodeling and may support the development of targeted anti-fibrotic and anti-inflammatory therapeutic approaches in ischemic heart disease.

Landmark clinical and translational studies investigating the relationship between ischemic heart disease, subclinical inflammation, and progression toward heart failure are summarized in Table 1.

### 3.3. Biomarkers of Subclinical Inflammation in Ischemic Heart Disease

Biomarkers have become increasingly important in the evaluation of patients with ischemic heart disease, particularly for identifying individuals at increased risk of developing heart failure. Given the central role of inflammatory processes in myocardial injury and remodeling, circulating inflammatory markers provide valuable insights into ongoing biological activity within the cardiovascular system. In this context, biomarkers of subclinical inflammation may enable the early detection of pathological changes before the onset of overt cardiac dysfunction [21,50].

Beyond their diagnostic utility, inflammatory biomarkers also contribute to risk stratification and may inform therapeutic decision-making. Elevated levels of specific inflammatory mediators have been consistently associated with adverse cardiovascular outcomes, including recurrent ischemic events, progressive myocardial remodeling, and the development of heart failure [18]. Among these, high-sensitivity C-reactive protein (hsCRP) remains one of the most extensively studied and clinically validated biomarkers of systemic inflammation in cardiovascular disease.

Key inflammatory and fibrosis-related biomarkers relevant to ischemic heart disease and heart failure are summarized in Table 2.

#### 3.3.1. High-Sensitivity C-Reactive Protein

High-sensitivity C-reactive protein (hsCRP) is a well-established biomarker of systemic inflammation and has been extensively investigated in the context of cardiovascular risk assessment. C-reactive protein is primarily synthesized by hepatocytes in response to stimulation by pro-inflammatory cytokines, particularly interleukin-6 (IL-6), which is released during inflammatory activation [51]. Advances in high-sensitivity assays have enabled the detection of very low circulating concentrations of CRP, allowing its use as a marker of low-grade inflammatory processes associated with atherosclerosis and cardiovascular disease.

Elevated hsCRP levels have been consistently associated with an increased risk of adverse cardiovascular outcomes. Large-scale epidemiological studies have demonstrated that individuals with higher hsCRP concentrations are more likely to experience myocardial infarction, stroke, or cardiovascular death compared with those with lower levels [23]. Furthermore, hsCRP has been shown to predict recurrent ischemic events in patients with established coronary artery disease, suggesting that persistent inflammatory activation contributes to ongoing vascular injury and plaque instability [52].

Beyond its role in atherosclerotic disease, hsCRP has also been linked to the development and progression of heart failure. Chronic systemic inflammation contributes to myocardial remodeling, endothelial dysfunction, and microvascular impairment—processes that ultimately lead to progressive deterioration of cardiac function [53]. Consequently, hsCRP measurement provides valuable insight into the inflammatory burden of patients with ischemic heart disease and may help identify individuals at increased risk of future cardiac dysfunction.

The clinical relevance and prognostic implications of hsCRP in ischemic heart disease are summarized in Table 2.

#### 3.3.2. Galectin-3

Galectin-3 has emerged as an important biomarker reflecting the interplay between inflammation and myocardial fibrosis in cardiovascular disease. This molecule is a β-galactoside-binding lectin primarily secreted by activated macrophages and other inflammatory cells in response to tissue injury. Through its regulatory role in immune and fibrotic pathways, galectin-3 promotes the activation of cardiac fibroblasts and stimulates collagen synthesis within the myocardial extracellular matrix [54,55].

Experimental and clinical studies have demonstrated that increased galectin-3 expression is closely associated with the development of myocardial fibrosis and structural remodeling. By promoting fibroblast proliferation and collagen deposition, galectin-3 contributes to progressive myocardial stiffening and impaired ventricular relaxation—key processes in the pathophysiology of heart failure [56]. In addition, galectin-3 has been implicated in the regulation of inflammatory signaling pathways and may act as a mediator linking chronic immune activation to structural alterations within the myocardium.

Circulating galectin-3 levels have therefore attracted considerable interest as a potential biomarker for risk stratification in patients with cardiovascular disease. Elevated concentrations have been associated with adverse clinical outcomes, including an increased risk of hospitalization and mortality in patients with heart failure. Furthermore, galectin-3 levels appear to correlate with the extent of myocardial remodeling and fibrosis, suggesting that this biomarker may provide insight into the underlying structural changes occurring during disease progression [45].

Given its involvement in both inflammatory and fibrotic pathways, galectin-3 is increasingly recognized as a valuable indicator of ongoing myocardial remodeling in patients with ischemic heart disease. Measurement of this biomarker may facilitate the early identification of patients at risk of developing heart failure and could potentially guide therapeutic strategies aimed at limiting myocardial fibrosis. The role of galectin-3 as a biomarker of myocardial fibrosis and its prognostic implications are summarized in Table 2.

#### 3.3.3. Cancer Antigen 125 (CA125)

Cancer antigen 125 (CA125), also known as mucin-16, was originally identified as a tumor marker used in the diagnosis and monitoring of ovarian cancer. However, accumulating evidence has demonstrated that CA125 also reflects systemic inflammatory activity and fluid congestion in patients with cardiovascular disease [47].

In the context of heart failure, elevated CA125 levels appear to result from activation of mesothelial cells lining the pericardial, pleural, and peritoneal cavities. Mechanical stress induced by fluid accumulation, together with inflammatory stimulation, promotes increased synthesis and release of this glycoprotein into circulation [57]. Consequently, circulating CA125 concentrations have been shown to correlate with the degree of systemic congestion and fluid overload in patients with heart failure.

Several clinical studies have demonstrated that elevated CA125 levels are associated with markers of disease severity, including increased filling pressures, pleural effusion, and pericardial inflammation [58]. In addition, CA125 has been linked to neurohormonal activation and inflammatory signaling pathways, further supporting its role as a biomarker reflecting the complex interplay between hemodynamic stress and systemic inflammation [59].

Importantly, CA125 has also demonstrated significant prognostic value in patients with cardiovascular disease. Higher circulating levels have been associated with an increased risk of hospitalization and mortality in heart failure populations, suggesting that this biomarker may provide complementary information to traditional cardiac markers [48]. For this reason, CA125 is increasingly being investigated as a useful tool for monitoring congestion and guiding therapeutic strategies in patients with heart failure related to ischemic heart disease.

### 3.4. Advanced Imaging in Inflammatory Cardiomyopathy

#### 3.4.1. Strain Echocardiography

Speckle-tracking echocardiography has emerged as a valuable imaging modality for the detailed assessment of myocardial function beyond the capabilities of conventional echocardiography. By enabling quantitative analysis of myocardial deformation, this technique allows the detection of subtle abnormalities in cardiac mechanics that may not yet be reflected by changes in left ventricular ejection fraction.

One of the most clinically relevant parameters derived from this method is global longitudinal strain (GLS). Multiple studies have demonstrated that GLS can identify early myocardial dysfunction in patients with ischemic heart disease, even in the presence of preserved left ventricular ejection fraction (LVEF). Owing to its high sensitivity, strain imaging is increasingly utilized for the detection of subclinical myocardial injury and for monitoring disease progression and therapeutic response [49,60,61].

Reduced GLS has been consistently associated with adverse cardiovascular outcomes, even in patients with preserved LVEF.

#### 3.4.2. Cardiac Magnetic Resonance Imaging

Cardiac magnetic resonance imaging (CMR) is widely regarded as the gold standard for the non-invasive assessment of myocardial structure and tissue characterization. Its ability to provide high-resolution anatomical and functional information makes it particularly valuable in the evaluation of both inflammatory and ischemic cardiomyopathies.

Advanced CMR techniques, including T1 and T2 mapping, enable the detection of diffuse myocardial abnormalities such as interstitial fibrosis, myocardial edema, and active inflammatory processes. In addition, late gadolinium enhancement (LGE) imaging allows the precise visualization of focal areas of myocardial scarring, which are commonly observed following ischemic injury.

Collectively, these imaging modalities provide comprehensive insights into the structural and functional consequences of chronic inflammatory activation in ischemic heart disease, thereby improving the understanding of disease progression and refining risk stratification strategies [62,63,64].

**Figure 2:**
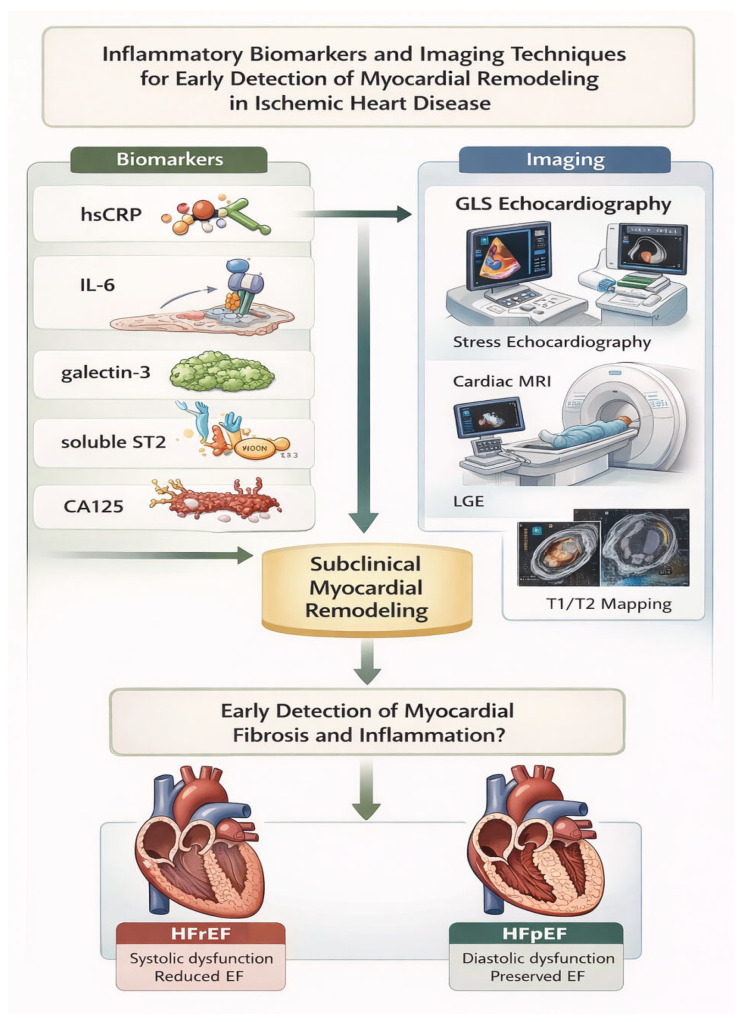
Inflammatory Biomarkers and Imaging Techniques.

A summary of representative clinical studies highlighting the diagnostic and prognostic value of advanced imaging modalities in ischemic heart disease is provided in Table 3. The complementary role of CMR and strain echocardiography in the assessment of myocardial inflammation and fibrosis is illustrated in Figure 2.

## 4. Discussion

Subclinical inflammation has emerged as a key contributor to the development and progression of ischemic heart disease (IHD) and its transition toward heart failure (HF). Accumulating evidence from randomized clinical trials, observational cohort studies, and biomarker analyses indicates that inflammatory pathways play a central role not only in the initiation and progression of atherosclerosis but also in post-ischemic myocardial remodeling and the subsequent development of HF [7,22,28].

One of the most important concepts highlighted by recent clinical research is the presence of residual inflammatory risk in patients with established cardiovascular disease. Even in the context of optimal lipid-lowering therapy and contemporary secondary prevention strategies, a substantial proportion of patients continue to exhibit elevated inflammatory markers, particularly high-sensitivity C-reactive protein (hs-CRP). This persistent inflammatory state has been consistently associated with an increased risk of major adverse cardiovascular events (MACE), including myocardial infarction, stroke, and HF hospitalization [23,27]. Randomized clinical trials investigating anti-inflammatory strategies have provided further support for the inflammatory hypothesis of atherosclerosis. Interventions targeting specific inflammatory pathways have demonstrated that modulation of inflammation can significantly reduce cardiovascular risk independently of lipid levels [28]. However, not all anti-inflammatory therapies have produced favorable results, suggesting that only certain inflammatory pathways play a dominant role in cardiovascular pathology [20].

Inflammation contributes to several key mechanisms involved in the progression from ischemic injury to cardiac dysfunction. Following myocardial ischemia or infarction, activation of the innate immune system triggers the release of inflammatory cytokines such as interleukin-1β (IL-1β), interleukin-6 (IL-6), and tumor necrosis factor-α (TNF-α) [8,19]. These mediators stimulate leukocyte recruitment, endothelial activation, and oxidative stress, which collectively contribute to further myocardial injury [13,15]. While the inflammatory response is essential for tissue repair in the acute phase, persistent low-grade inflammation may promote maladaptive processes such as extracellular matrix remodeling, fibroblast activation, and myocardial fibrosis [17,39]. These structural changes ultimately lead to ventricular remodeling, progressive decline in systolic and diastolic function, and the development of clinical heart failure [40].

The role of inflammatory biomarkers in identifying patients at risk of disease progression has been increasingly recognized. Traditional inflammatory markers such as hs-CRP and IL-6 have been widely studied and consistently associated with adverse cardiovascular outcomes [23,26]. Elevated hs-CRP levels have been shown to predict incident HF in large population-based cohorts, suggesting that systemic inflammation may precede the development of overt cardiac dysfunction [53]. Similarly, increased IL-6 concentrations have been associated with greater risk of HF hospitalization and mortality in patients with coronary artery disease [26]. These findings support the hypothesis that inflammation acts as a driver of disease progression rather than merely reflecting the severity of underlying cardiovascular pathology.

In addition to traditional inflammatory markers, novel biomarkers such as galectin-3 and cancer antigen 125 (CA125) have gained increasing attention in recent years. Galectin-3 is a β-galactoside-binding lectin secreted by activated macrophages and is involved in processes such as fibroblast proliferation, collagen deposition, and extracellular matrix remodeling [54,55,56]. Elevated galectin-3 levels have been associated with myocardial fibrosis, ventricular remodeling, and increased mortality in patients with HF [45]. Importantly, galectin-3 appears to reflect a key biological pathway linking inflammation with myocardial fibrosis, which is a fundamental mechanism underlying the progression from ischemic cardiomyopathy to overt heart failure.

Similarly, CA125 has emerged as a biomarker that reflects the complex interaction between inflammation, congestion, and neurohormonal activation in HF. Although originally used as a tumor marker, CA125 is produced by mesothelial cells in response to mechanical stress and inflammatory cytokines [47,59]. Elevated levels have been observed in patients with acute and chronic HF and have been associated with worse clinical outcomes, including increased mortality and HF-related hospitalizations [48]. Recent studies suggest that CA125 may also reflect systemic inflammatory activation and may amplify the effects of inflammatory cytokines such as IL-6 [57]. Consequently, CA125 may serve as a valuable biomarker for identifying patients with subclinical congestion and inflammatory activation, both of which contribute to HF progression.

Another important aspect highlighted by current evidence is the role of inflammation in adverse ventricular remodeling following myocardial infarction. Persistent inflammatory activation may lead to cardiomyocyte apoptosis, increased oxidative stress, and activation of profibrotic signaling pathways [15,17]. These processes result in progressive ventricular dilation, reduced contractile function, and eventual development of ischemic cardiomyopathy [40]. Biomarkers reflecting inflammatory and fibrotic activity may therefore provide important prognostic information and may help identify patients who could benefit from early therapeutic interventions aimed at preventing HF development.

In addition to the well-characterized cytokine-mediated inflammatory pathways, increasing evidence suggests that autoimmune mechanisms may also contribute to the persistence and amplification of myocardial inflammation following ischemic injury. Myocardial ischemia and necrosis lead to the release of intracellular cardiac antigens, which can subsequently trigger the production of anti-cardiac autoantibodies directed against structural and contractile proteins of cardiomyocytes. These autoantibodies have been detected in a substantial proportion of patients following myocardial infarction and are believed to play an important role in sustaining secondary inflammatory responses within the myocardium. Through activation of complement pathways and immune-mediated cellular injury, anti-cardiac autoantibodies may contribute to ongoing myocardial damage, adverse remodeling, and progressive ventricular dysfunction [67,68].

In this context, the development of secondary myocarditis has been increasingly recognized as a potential complication following myocardial infarction. Post-ischemic myocardial injury may initiate an immune-mediated inflammatory reaction characterized by infiltration of immune cells, activation of resident macrophages, and persistence of inflammatory signaling within the myocardium. This inflammatory process resembles features of myocarditis and may represent an important component of the broader inflammatory cascade associated with ischemic heart disease. Importantly, the presence of secondary myocarditis has been associated with more extensive myocardial injury, impaired ventricular function, and a significantly increased risk of progression to heart failure [8,69].

Taken together, these observations suggest that autoimmune mechanisms and post-infarction myocardial inflammation may represent additional contributors to the complex interplay between ischemic injury and chronic inflammatory activation. Recognition of these processes may provide further insight into the heterogeneity of inflammatory responses observed in ischemic heart disease and could open new perspectives for targeted immunomodulatory strategies aimed at preventing adverse cardiac remodeling and heart failure progression.

Beyond circulating biomarkers and advanced imaging parameters, emerging evidence suggests that structural thoracic characteristics may also influence systemic inflammatory burden and cardiovascular risk.

Thoracic morphology has recently emerged as a potentially relevant, yet relatively underexplored, determinant of systemic inflammatory burden and cardiovascular risk. Structural characteristics such as chest wall configuration, thoracic cage dimensions, and the distribution of intrathoracic adipose tissue may influence cardiopulmonary mechanics as well as local immune responses. In particular, increased intrathoracic or mediastinal adipose tissue has been associated with a pro-inflammatory milieu characterized by elevated cytokine production, oxidative stress, and endothelial dysfunction—mechanisms that are known to contribute to the development and progression of cardiovascular disease. Recent evidence indicates that certain structural thoracic features, such as an increased anterior–posterior chest diameter assessed by the modified Haller index, may be linked to early cardiopulmonary changes and subtle cardiac dysfunction, suggesting their potential value as noninvasive markers of early cardiovascular involvement [70,71,72,73].

Furthermore, variations in thoracic geometry may influence cardiac filling dynamics, pulmonary vascular interactions, and overall cardiopulmonary coupling. These structural factors could indirectly modulate hemodynamic stress and inflammatory activation, thereby contributing to the complex pathophysiological processes linking ischemic heart disease to myocardial remodeling and heart failure.

From a translational perspective, the integration of thoracic morphological parameters obtainable through routine imaging modalities such as computed tomography or cardiac magnetic resonance into cardiovascular risk assessment models may provide incremental value beyond traditional biomarkers of inflammation. Quantitative assessment of thoracic adipose tissue and thoracic structural characteristics could therefore represent an additional tool for identifying individuals with increased inflammatory burden and subclinical cardiovascular risk.

Within the broader framework of precision cardiovascular medicine, multidimensional approaches that combine imaging-derived structural parameters with circulating biomarkers and functional imaging markers may facilitate earlier identification of high-risk individuals. Such strategies could ultimately support more personalized preventive and therapeutic interventions aimed at reducing inflammatory activation and preventing adverse cardiac remodeling. Nevertheless, further prospective studies are needed to validate thoracic morphology as a reproducible and clinically actionable marker within inflammatory and cardiovascular risk stratification frameworks [72,73,74,75,76].

Despite the growing body of evidence supporting the role of inflammation in cardiovascular disease, several challenges remain. First, the inflammatory response involved in cardiovascular pathology is highly complex and involves multiple overlapping signaling pathways. As a result, targeting a single inflammatory mediator may not be sufficient to produce significant clinical benefits in all patient populations. Second, inflammatory biomarkers may reflect different pathophysiological processes depending on the clinical context, making their interpretation challenging. Finally, the optimal therapeutic strategies for modulating inflammation in cardiovascular disease remain to be fully established.

Future research should focus on improving the understanding of inflammatory phenotypes in patients with ischemic heart disease and identifying those individuals who are most likely to benefit from targeted anti-inflammatory therapies. Advances in biomarker profiling, proteomics, and precision medicine approaches may facilitate the identification of specific inflammatory pathways involved in individual patients. Such strategies could ultimately enable the development of personalized therapeutic interventions aimed at preventing the transition from ischemic heart disease to heart failure.

## 5. Conclusions

In conclusion, current evidence strongly supports the concept that subclinical inflammation represents a fundamental pathophysiological link between ischemic heart disease and the development of heart failure. Biomarkers such as hs-CRP, IL-6, galectin-3, and CA125 provide valuable insights into the inflammatory and fibrotic processes underlying cardiac remodeling and may improve risk stratification in patients with coronary artery disease. A better understanding of these mechanisms may open up new avenues for therapeutic intervention and ultimately contribute to improved prevention of heart failure in patients with ischemic heart disease.

## Figures and Tables

**Table 1:** Landmark studies linking ischemic heart disease, inflammation, and heart failure.

Study	Study Design	Mechanism Investigated	Key Findings	Clinical Implications
Frangogiannis et al., 2014 [8]	Review and mechanistic studies	Inflammatory response after myocardial injury	Demonstrated that post-ischemic inflammation regulates myocardial repair and remodeling	Established inflammation as a central component of cardiac healing
Frangogiannis et al., 2002 [9]	Experimental and mechanistic studies	Post-infarction inflammatory cascade	Myocardial infarction triggers a coordinated inflammatory response necessary for tissue repair.	Provided early mechanistic understanding of inflammatory signaling in MI
Timmers et al., 2012 [10]	Experimental studies	Innate immune activation	Activation of innate immune pathways contributes to myocardial injury and remodeling.	Highlighted importance of immune signaling in reperfusion injury.
Nahrendorf et al., 2010 [11]	Experimental imaging study	Monocyte recruitment	Monocytes play key roles in infarct inflammation and tissue repair.	Identified immune cell dynamics during cardiac healing.
Nahrendorf et al., 2007 [12]	Experimental study	Monocyte subset recruitment	Sequential recruitment of inflammatory and reparative monocytes regulates infarct healing.	Demonstrated temporal regulation of immune responses.
Frangogiannis 2012 [13]	Mechanistic study	Regulation of cardiac inflammation	Inflammatory signaling controls myocardial repair and fibrosis after injury.	Provided insight into inflammatory regulation of cardiac remodeling.
Westman et al., 2016 [15]	Clinical and experimental study	Inflammation and ventricular remodeling	Persistent inflammation contributes to adverse ventricular remodeling after MI.	Identified inflammation as driver of HF progression after MI.
Libby, 2002 [16]	Pathophysiological study	Inflammation in atherosclerosis	Chronic inflammation plays a fundamental role in plaque formation and instability.	Established inflammatory basis of coronary artery disease.
Hansson, 2005 [18]	Clinical review	Immune mechanisms in CAD	Atherosclerosis is an immune-mediated inflammatory disease.	Reinforced concept of immune involvement in CAD.
Mann, 2002 [19]	Clinical heart failure studies	Cytokine activation	Elevated inflammatory cytokines associated with heart failure severity.	Demonstrated link between inflammation and HF progression.
Ridker, 2014 [21]	Epidemiological study	CRP and cardiovascular risk	Inflammation predicts cardiovascular risk independently of cholesterol.	Supported use of inflammatory biomarkers in risk assessment.
Ridker et al., 2002 [23]	Prospective cohort study	hsCRP and cardiovascular risk	Elevated hsCRP strongly predicts first cardiovascular events.	Established hsCRP as major biomarker.
Ridker, 2016 [7]	Translational research	Cytokine signaling	IL-1 and IL-6 pathways central in atherosclerotic inflammation.	Identified upstream therapeutic targets.
Abbate et al., 2020 [24]	Clinical and experimental studies	IL-1 signaling	IL-1 contributes to ventricular remodeling and cardiac dysfunction.	Demonstrated therapeutic potential of IL-1 inhibition.
Toldo and Abbate, 2018 [25]	Experimental studies	NLRP3 inflammasome	Inflammasome activation contributes to myocardial injury after MI.	Identified novel inflammatory pathway in cardiac injury.
Danesh et al., 2008 [26]	Meta-analysis	IL-6 and cardiovascular risk	Elevated IL-6 associated with increased coronary heart disease risk.	Highlighted role of cytokines in systemic inflammation.
Emerging Risk Factors Collaboration [27]	Prospective cohort analysis	CRP and coronary risk	CRP levels associated with long-term cardiovascular outcomes.	Confirmed prognostic value of inflammatory biomarkers.
Ridker et al., 2017 [28]—CANTOS	Randomized clinical trial	IL-1β inhibition	Anti-inflammatory therapy reduced recurrent cardiovascular events.	Provided proof that targeting inflammation reduces CV risk.
Sharma et al., 2000 [29]	Clinical study	Cytokine-mediated myocardial dysfunction	Inflammatory mediators contribute to cardiac dysfunction in HF.	Highlighted role of systemic inflammation in HF.
Mann, 2002 [19]	Clinical and mechanistic studies	TNF-α signaling	TNF-α exerts negative inotropic effects and contributes to myocardial dysfunction.	Established cytokines as mediators of HF progression.

**Table 2:** Inflammatory biomarkers involved in ischemic heart disease and heart failure.

Study	Study Design	Biomarker	Key Findings	Clinical Relevance
Ridker et al., 2002 [23]	Prospective Cohort	High-sensitivity C-reactive protein (hsCRP)	Elevated hsCRP predicts myocardial infarction and stroke.	Widely used biomarker for cardiovascular risk stratification.
Abbate et al., 2020 [24]	Mechanistic and clinical studies	Interleukin-1β (IL-1β)	IL-1 signaling contributes to myocardial inflammation and ventricular remodeling.	Potential therapeutic target in cardiovascular disease.
Danesh et al., 2008 [26]	Meta-analysis of prospective studies	Interleukin-6 (IL-6)	Higher IL-6 levels associated with increased coronary heart disease risk.	Marker of systemic inflammation and cardiovascular risk.
Mann et al., 2002 [19]	Clinical studies	Tumor necrosis factor-α (TNF-α)	Elevated TNF-α associated with disease severity and myocardial dysfunction.	Reflects inflammatory activation in heart failure.
Lok et al., 2010 [45]	Clinical heart failure cohort	Galectin-3	Galectin-3 associated with myocardial fibrosis and adverse outcomes.	Prognostic biomarker in heart failure.
de Boer et al., 2011 [46]	Biomarker study	Galectin-3	Elevated galectin-3 correlates with cardiac remodeling and mortality.	Useful for HF risk stratification.
Januzzi et al., 2017 [44]	Multicenter HF study	Soluble ST2	Elevated sST2 predicts worse outcomes in heart failure.	Biomarker of myocardial stress and fibrosis.
D’Aloia et al., 2003 [47]	Heart failure cohort	Cancer antigen 125 (CA125)	CA125 levels correlate with congestion and disease severity.	Marker of fluid overload.
Núñez et al., 2014 [48]	Prospective HF study	Cancer antigen 125 (CA125)	Elevated CA125 predicts hospitalization and mortality.	Useful biomarker for prognosis and congestion monitoring.
Spinale et al., 2007 [40]	Experimental and clinical studies	Matrix metalloproteinases (MMPs)	MMP dysregulation contributes to extracellular matrix remodeling and ventricular dilation.	Indicator of pathological myocardial remodeling.

**Table 3:** Imaging modalities for detecting inflammatory myocardial injury and remodeling.

Study	Study Design	Imaging Technique	Key Findings	Clinical Relevance
Yingchoncharoen et al., 2013 [60]	Meta-analysis	Speckle-tracking echo-cardiography	Global longitudinal strain detects early myocardial dysfunction before LVEF decline.	Sensitive marker of subclinical myocardial injury.
Kalam et al., 2014 [49]	Systematic review and meta-analysis	Strain echo-cardiography	GLS predicts cardiovascular outcomes independently of LVEF.	Prognostic imaging parameter.
Sicari et al., 2008 [61]	Multicenter study	Stress echo-cardiography	Stress echocardiography detects inducible ischemia and myocardial viability.	Non-invasive evaluation of ischemia.
Kim et al., 2000 [65]	Clinical imaging study	Cardiac magnetic resonance (CMR)	Contrast-enhanced MRI identifies myocardial infarction and scar tissue.	Gold standard for myocardial tissue characterization.
Wu et al., 2001 [66]	Clinical imaging study	Late gadolinium enhancement (LGE) CMR	LGE extent correlates with infarct size and functional recovery.	Important for risk stratification and prognosis.
Puntmann et al., 2016 [62]	Prospective cohort study	T1 mapping CMR	Native T1 mapping detects diffuse myocardial fibrosis and predicts outcomes.	Early identification of myocardial remodeling.
Ferreira et al., 2013 [63]	Imaging study	T2 mapping CMR	T2 mapping detects myocardial edema and inflammation.	Useful for diagnosing inflammatory cardiomyopathies.

## Data Availability

No new data were created or analyzed in this study. Data sharing is not applicable to this article.
